# The Multidisciplinary Management of Placenta Accreta Spectrum: A Comprehensive Series of Eight Cases From a Tertiary Care Center

**DOI:** 10.7759/cureus.96116

**Published:** 2025-11-05

**Authors:** Rashida Ali, Nimish Tutwala, Mena Abdalla

**Affiliations:** 1 Obstetrics and Gynaecology, Princess Royal University Hopsital, London, GBR; 2 Obstetrics and Gynaecology, Topiwala National Medical College & B. Y. L. Nair Charitable Hospital, Mumbai, IND; 3 Medical Education, Queen's University Belfast, Belfast, GBR; 4 Obstetrics and Gynaecology, King's College Hospital NHS Foundation Trust, London, GBR

**Keywords:** cesarean hysterectomy, maternal morbidity, multidisciplinary care, obstetric hemorrhage, palcenta accreta, uterine artery embolization

## Abstract

Background and objective

Placenta accreta spectrum (PAS) is a severe obstetric complication associated with significant maternal morbidity and mortality. As cesarean delivery rates continue to rise, the prevalence of PAS is increasing, creating a growing challenge for healthcare systems. This study aimed to present a comprehensive analysis of the multidisciplinary management of PAS in a tertiary care center.

Methods

We performed a retrospective case series of eight consecutive cases of PAS managed at a tertiary care government hospital between January 2023 and December 2024 (24 months). Data were collected from hospital records, including patient demographics, clinical presentation, imaging findings, surgical management, and maternal outcomes. All cases were managed by a dedicated multidisciplinary team.

Results

The study included eight patients with a mean age of 29.6 years. All patients had undergone at least one previous cesarean section. The mean gestational age at diagnosis was 32 weeks, and the mean gestational age at delivery was 36.4 weeks. Six patients underwent cesarean hysterectomy, one had a hysterotomy with uterine artery embolization (UAE), and one had a conservative management approach. The mean estimated blood loss was 2.1 litres. There were no maternal deaths, and the most frequent complication observed was bladder injury (n=2).

Conclusions

This study highlights the importance of a multidisciplinary approach in the management of PAS. Early diagnosis, individualized management strategies, and a coordinated team of specialists can lead to favorable maternal outcomes, even in complex cases. Our results highlight the importance of establishing specialized centers of excellence for managing PAS.

## Introduction

Placenta accreta spectrum (PAS) encompasses a range of abnormal placental adherence conditions, including placenta accreta, increta, and percreta, characterized by varying degrees of myometrial invasion [1]. Over the past three decades, the global incidence has risen sharply, from about 0.12 per 1,000 deliveries in the 1970s to 3.0 per 1,000 deliveries today, largely due to the increasing prevalence of cesarean deliveries [2]. In India, the reported incidence is 0.9 per 1000 deliveries, though this likely represents an underestimate due to underreporting and diagnostic challenges [3]. The rising incidence of PAS, driven by increasing cesarean section rates, poses a major challenge to maternal health services globally, highlighting the need for specialized centers and standardized, evidence-based management protocols.

The pathophysiology of PAS involves defective decidualization at the endometrial-myometrial interface, typically occurring at sites of previous uterine surgery. This defect allows abnormal trophoblastic invasion beyond the normal boundaries, leading to varying degrees of placental adherence to or invasion through the myometrium [4]. The primary risk factors include previous cesarean delivery, placenta previa, advanced maternal age, multiparity, and history of uterine curettage or other intrauterine procedures [5].

PAS represents a leading cause of peripartum hysterectomy and is associated with significant maternal morbidity and mortality, primarily due to massive obstetric hemorrhage [6]. The successful management of the condition requires early antenatal diagnosis, delivery in a tertiary care center with appropriate resources, and a coordinated multidisciplinary team approach [7]. The standard management involves planned cesarean hysterectomy with the placenta left in situ, though conservative approaches may be considered in carefully selected cases [8].

This comprehensive case series presents our experience managing eight consecutive cases of PAS at a tertiary care government hospital, highlighting the diversity of clinical presentations and the effectiveness of a standardized multidisciplinary approach in achieving favorable maternal outcomes.

## Materials and methods

Study design

This retrospective case series analyzes eight consecutive patients diagnosed with PAS and managed at a tertiary care government hospital between January 2023 and December 2024. Cases were identified through hospital records and included all patients with a confirmed PAS diagnosis based on clinical, imaging, and histopathological criteria.

Study population and sample size

The study population included all consecutive patients who were diagnosed with PAS during the study period. A total of eight patients met the predefined inclusion criteria and were consequently enrolled in this case series. Of these, five (62.5%) were registered cases, and three (37.5%) were referrals from other centers. All cases were managed at our tertiary care center by a dedicated multidisciplinary team to ensure optimal maternal and fetal outcomes. Our hospital is a tertiary referral center with 200 obstetric beds and approximately 7,000 deliveries per year.

Inclusion and exclusion criteria

The study enrolled pregnant women diagnosed with PAS either through ultrasonography, MRI, or as an intraoperative finding. Only patients who delivered at our tertiary care government hospital between January 2023 and December 2024 were deemed eligible. A confirmed diagnosis of PAS was established through clinical evaluation, imaging results, and, when applicable, histopathological examination. Additionally, only patients with complete medical records available for review and those who provided written informed consent for the publication of their clinical data were included in the analysis.

Patients were excluded from the study if their medical records were incomplete, if they declined to provide consent for the publication of clinical information, or if PAS was suspected but not confirmed on final diagnosis.

Study measures

Data Collection

Data collection included demographic characteristics, obstetric history, clinical presentation, imaging findings, surgical details, blood transfusion requirements, complications, and maternal outcomes. All data were extracted from electronic medical records and surgical case notes by the research team.

Antenatal Evaluation

All patients underwent standardized antenatal evaluation, including detailed obstetric history, clinical examination, and imaging studies. Ultrasound examination was performed using standardized protocols to assess placental location, thickness of myometrium at the cesarean scar site, presence of placental lacunae, and vascular abnormalities. All scans were performed and reported by a dedicated team of radiologists with expertise in PAS imaging. While a formal ultrasound risk scoring system was not uniformly applied, reports were detailed and descriptive. MRI was utilized in selected cases for further characterization of placental invasion depth and involvement of adjacent organs, particularly in cases of posterior previa or suspected percreta.

Multidisciplinary Management Protocol

Management followed a standardized multidisciplinary protocol involving a core team of senior obstetricians (each with >10 years of experience), anesthesiologists, interventional radiologists, urologists, and intensive care specialists. All deliveries were planned in the main operating theater, which is equipped for major surgeries, unlike the labor room operating theater, which is reserved for minor procedures. The main OR has immediate access to blood bank facilities, the ICU, and emergency surgical services. Massive transfusion protocols were activated for all cases, with cross-matched blood products readily available. The cross-match to transfusion (CT) ratio was 1.5:1.

Surgical Approach

The surgical approach was individualized based on the severity of placental invasion, patient preferences, and intraoperative findings. Options included cesarean hysterectomy with placenta left in situ, hysterotomy with prophylactic uterine artery embolization (UAE), or conservative management with close monitoring in selected cases. The choice of skin incision (Pfannenstiel or midline vertical) was at the discretion of the primary surgeon, based on the anticipated complexity of the case.

Ethics statement

The study was conducted in accordance with the Declaration of Helsinki and received approval from the Institutional Ethics Committee of Grant Government Medical College, Mumbai. Written informed consent was obtained from all patients for publication of their clinical data and images.

Statistical analysis

Descriptive statistics were used to summarize patient demographics, clinical characteristics, and outcomes. Continuous variables were expressed as means with ranges, while categorical variables were presented as frequencies and percentages. Given the small sample size and descriptive nature of this case series, no inferential statistical analyses were performed.

## Results

A summary of the eight cases is presented in Table 1. The mean age of the patients was 29.6 years (range: 23-36 years). All patients had at least one prior cesarean delivery, with a mean of 1.75 previous cesarean sections. The mean gestational age at diagnosis of PAS was 32 weeks (range: 20-36 weeks). The mean gestational age at delivery was 36.4 weeks (range: 22-38 weeks). The mean estimated blood loss was 2.1 L (range: 0.6-3.0 L), and the mean operative time was 3.1 hours (range: 1-4 hours). There were no maternal deaths.

**Table 1:** Summary of all eight cases Outcome criteria: excellent - no major complications, rapid recovery; good - manageable complications, full recovery GA: gestational age; CS: cesarean section; OH: obstetric hysterectomy; UAE: uterine artery embolization; PRBC: packed red blood cells; FFP: fresh frozen plasma; RDP: random donor platelets; HPE: histopathological examination; AIP: FIGO-AIP (abnormally invasive placenta) Grade

Case	Age, years	Gravidity	Previous CS	GA at diagnosis (weeks)	GA at delivery (weeks)	Pre-op diagnosis	Post-op (HPE) diagnosis	AIP grade and topography	Management	Operative time (hours)	Blood loss (L)	Transfusion (PRBC/FFP/RDP)	ICU days	Hospital stay (days)	Outcome	Morbidity
1	28	G3P2	2	36	36	Accreta	Accreta	Grade 1, anterior	Emergency OH	2.5	2.5	4/2/0	0	8	Excellent	None
2	32	G3P1	1	32	38	Accreta	Accreta	Grade 1, posterior	Emergency OH	1	2.0	3/0/0	0	7	Excellent	None
3	23	G1P0	0	20	22	Increta	Increta	Grade 2, anteroinferior	UAE + hysterotomy	1.5	0.6	None	0	4	Fertility preserved	None
4	29	G2P1	1	28	38	Percreta	Percreta	Grade 3, anterior with bladder invasion	Elective OH + bladder repair	3.5	1.8	2/2/0	0	10	Excellent	Bladder injury
5	31	G3P2	2	32	38	Percreta	Percreta	Grade 3, anterior with bladder invasion	Elective OH	4	2.2	4/4/0	5	15	Excellent	Prolonged ICU stay
6	34	G3P2	2	37	37	Percreta	Percreta	Grade 3, anterior with bladder invasion	Emergency OH + bladder repair	4	3.0	4/4/4	2	12	Good	Bladder injury, TRALI
7	36	G5P3	3	36	36	Percreta	Percreta	Grade 3, anterior	Elective OH + bladder repair	3.5	2.4	4/4/0	7	14	Excellent	Prolonged ICU stay
8	30	G4P2	2	36	36	Increta	Increta	Grade 2, posterior	Emergency OH	2.5	1.5	2/2/0	1	8	Excellent	None

Case presentations

Case 1: Placenta Accreta With Complete Previa

A 28-year-old third gravida with two previous cesarean sections presented at 36 weeks of gestation with abdominal pain and mild vaginal bleeding. Her obstetric history included two prior lower-segment cesarean sections at term with uncomplicated recoveries. Initial ultrasound examination revealed complete placenta previa covering the internal cervical os with loss of the clear zone and presence of multiple placental lacunae. Color Doppler imaging demonstrated increased vascularity at the placental-myometrial interface. MRI could not be performed because of the emergency nature of the presentation. Ultrasound findings were consistent with placenta accreta spectrum.

**Figure 1:**
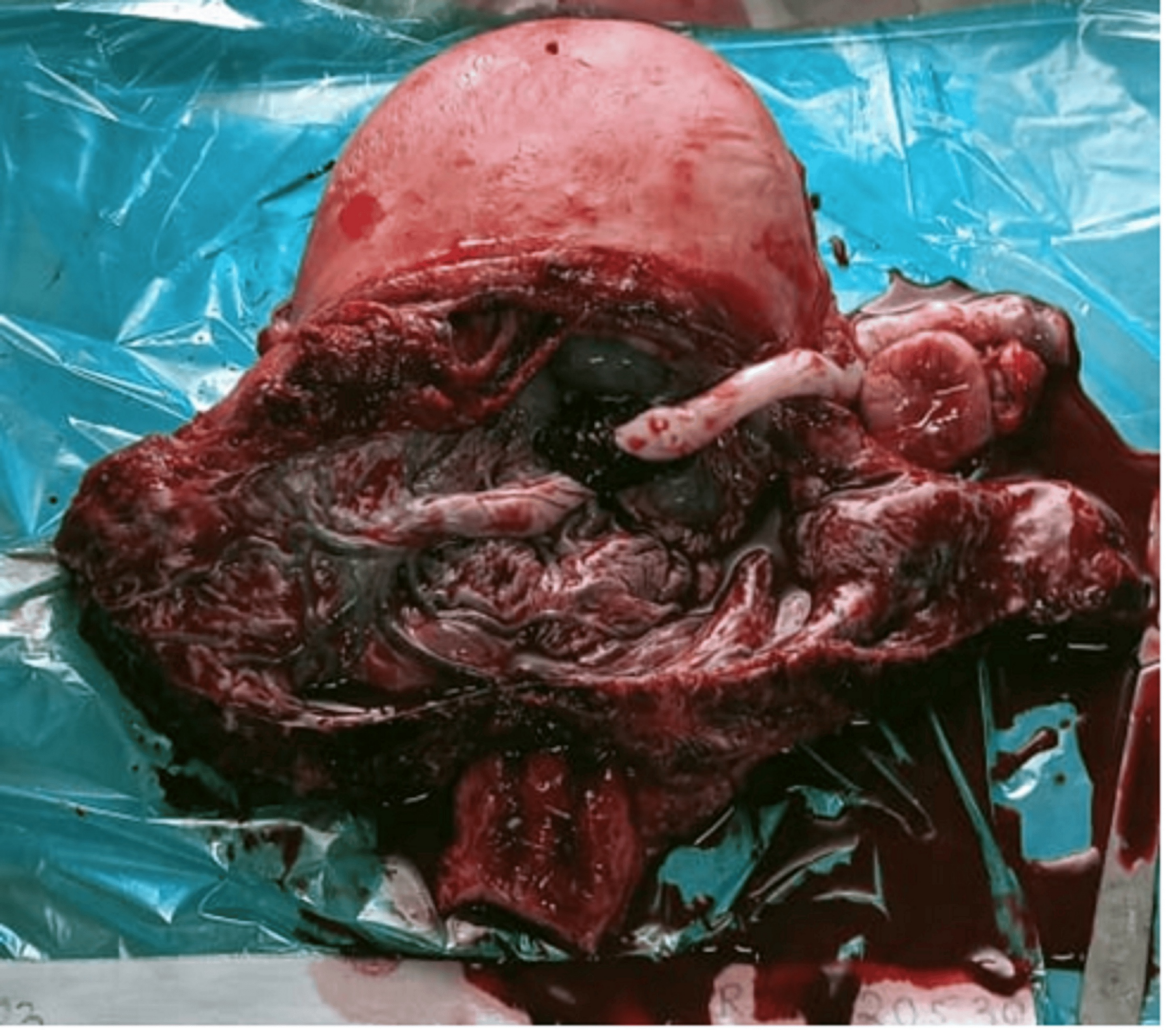
Hysterectomy specimen showing extensive neovascularization at the lower uterine segment Surgical specimen photograph demonstrating the characteristic “spaghetti-like” appearance of dilated vessels over the lower uterine segment and bladder serosa, a pathognomonic sign of placenta accreta spectrum requiring careful surgical planning

An emergency cesarean section was performed under spinal anesthesia due to the onset of active labor and escalating bleeding. Intraoperatively, extensive neovascularization was noted over the lower uterine segment and bladder serosa. A higher transverse uterine incision was made to avoid the highly vascular lower segment, and a healthy male infant weighing 2.8 kg was delivered. The placenta failed to separate spontaneously, and gentle attempts at manual removal resulted in torrential hemorrhage from the placental bed. An immediate decision for an obstetric hysterectomy was made to control bleeding. Total abdominal hysterectomy was performed, and the histopathology confirmed placenta accreta (AIP Grade 1). The patient required transfusion of four units of packed red blood cells and two units of fresh frozen plasma (FFP). Postoperative recovery was uncomplicated, and she was discharged on postoperative day eight (Figure 1).

Case 2: Placenta Accreta With Rapid Surgical Decision-Making

**Figure 2:**
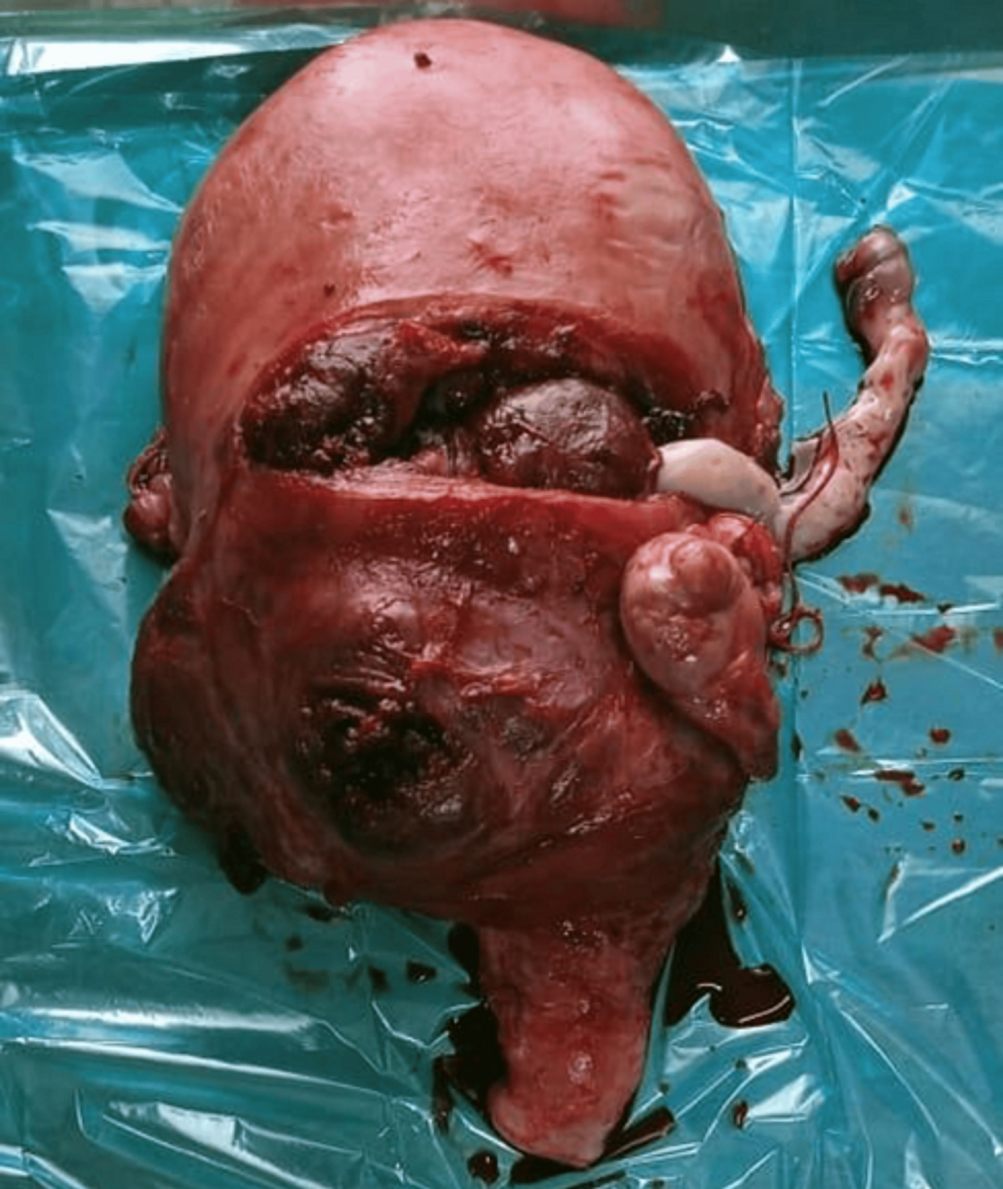
Surgical specimen of uterus demonstrating placenta accreta with firm adherence Gross pathological specimen showing the uterus with placenta firmly adherent to the myometrium. The placental tissue cannot be easily separated from the uterine wall, confirming the diagnosis of placenta accreta

A 32-year-old third gravida with one previous spontaneous abortion and one living child delivered by cesarean section was referred at 38 weeks of gestation with mild preeclampsia and scar tenderness. An antenatal ultrasound at 32 weeks had shown posterior placenta previa grade 2 with suspicious features of placental adherence. Follow-up imaging at 36 weeks confirmed progression to complete placenta previa with increased vascularity and loss of the clear zone. Emergency cesarean section was indicated due to increasing scar tenderness during latent labor and fetal heart rate abnormalities. Under combined spinal-epidural anesthesia, a transverse lower segment incision was made. The placenta was located posteriorly, extending into the lower segment with firm adherence to the myometrium. A healthy female infant weighing 3.1 kg was delivered. Partial placental separation was achieved manually, but complete removal by controlled cord traction was impossible. A prompt decision for an obstetric hysterectomy was made. The procedure was completed using the drop-down technique with systematic ligation of uterine vessels and careful dissection of the bladder. Histopathological examination confirmed placenta accreta (AIP Grade 1). The patient recovered uneventfully and was discharged on postoperative day seven (Figure 2).

Case 3: Second-Trimester Termination With Prophylactic Embolization

A 23-year-old primigravida with no previous uterine surgery presented at 22 weeks of gestation for termination of pregnancy due to fetal bilateral renal agenesis. Initial ultrasound at 20 weeks had revealed complete placenta previa grade 4 with concerning features. MRI confirmed focal placenta increta in the anteroinferior region (AIP Grade 2). Given the high risk of catastrophic hemorrhage and the patient’s strong desire for fertility preservation, a multidisciplinary team decision was made to proceed with prophylactic bilateral UAE before surgical intervention. The procedure was performed by interventional radiology. Hysterotomy was performed 24 hours after embolization to allow for ischemic changes to develop in the placental bed, thereby reducing the risk of hemorrhage. The placenta was removed with minimal bleeding, and the uterus was repaired in two layers using absorbable sutures. The patient was discharged on postoperative day four without requiring a blood transfusion.

Case 4: Placenta Percreta With Urological Intervention

**Figure 3:**
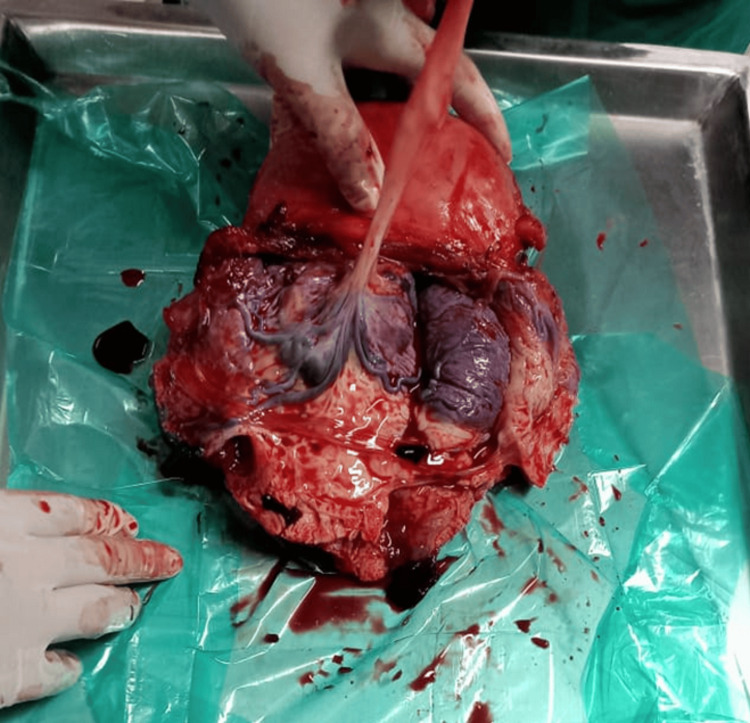
Post-hysterectomy specimen placenta percreta with bladder invasion An image demonstrating placenta percreta with evidence of placental invasion into the posterior bladder wall, crucial for surgical planning

A 29-year-old second gravida with one previous cesarean section was diagnosed with placenta percreta at 28 weeks of gestation. MRI at 34 weeks confirmed placenta percreta with focal areas of placental tissue extending through the anterior uterine wall and abutting the posterior bladder wall (AIP Grade 3). Elective cesarean section was planned at 38 weeks with urology and cardiothoracic surgery teams on standby. A Pfannenstiel incision was made, and a classical cesarean section was performed under general anesthesia. The placenta was left in situ, and the uterine incision was closed. During the subsequent hysterectomy, direct placental invasion into the bladder wall was revealed. The urological team performed a partial cystectomy to achieve complete removal of invaded tissue, followed by primary bladder repair and cystoscope-guided bilateral DJ stenting. The bladder was repaired in two layers using absorbable sutures. Methylene blue dye was used to confirm the absence of leakage. The bladder catheter was left in situ for 14 days. Histopathology confirmed placenta percreta with bladder invasion. The patient required prolonged ureteric stenting for six weeks. She was discharged on postoperative day 10 (Figure 3).

Case 5: Conservative Management of Placenta Percreta

**Figure 4:**
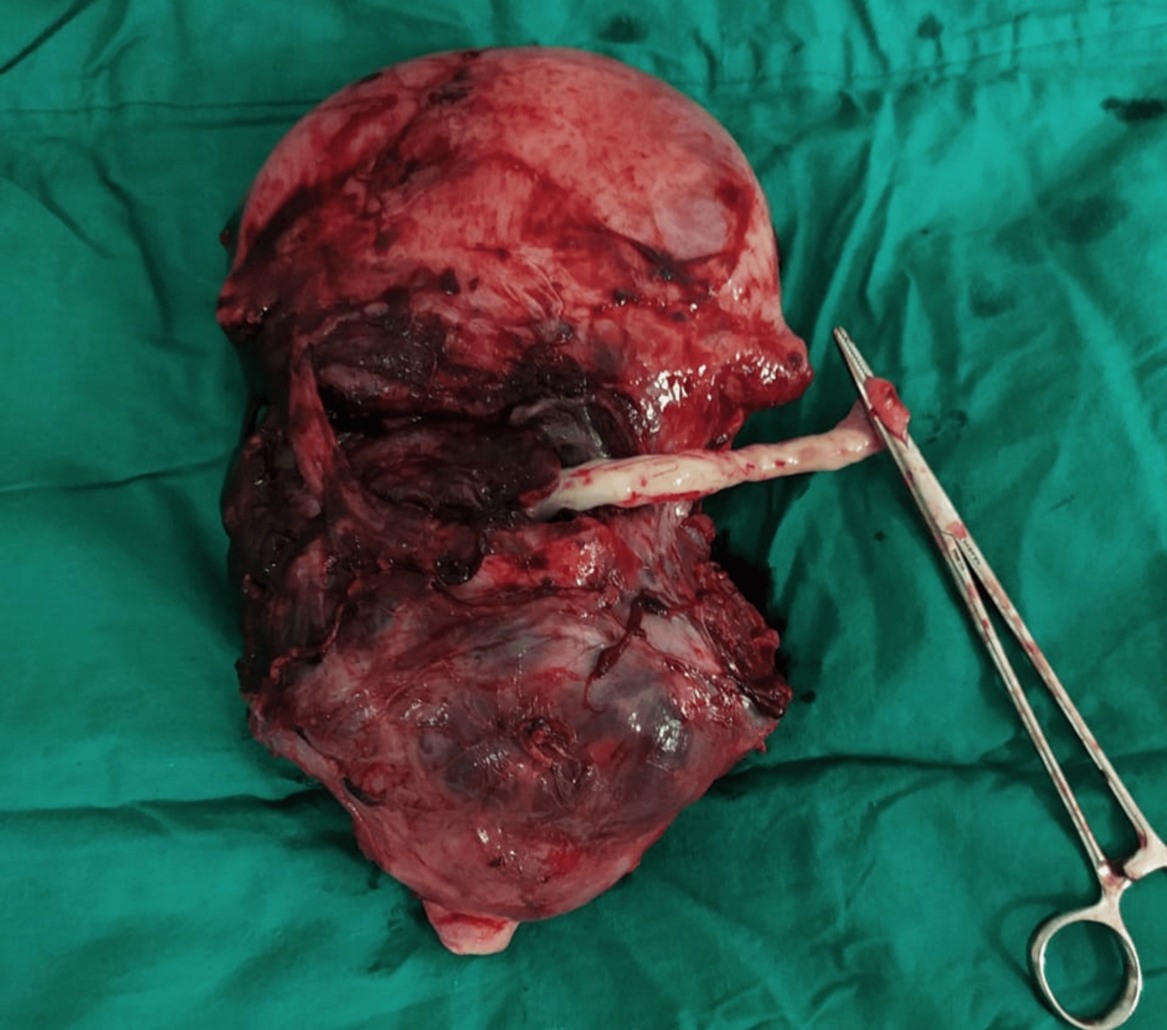
An image demonstrating placenta percreta with evidence of placental invasion into the posterior bladder wall, crucial for surgical planning A surgical specimen view showing the well-defined placenta attaches itself even more deeply into the muscle wall of the uterus

A 31-year-old third gravida with two previous cesarean sections was diagnosed with placenta percreta and bladder invasion at 32 weeks of gestation (AIP Grade 3). Given the early gestational age and stable clinical condition, a decision was made for conservative management with close monitoring to optimize fetal maturity. The patient was admitted for bed rest with continuous fetal and maternal monitoring, including twice-weekly cardiotocography (CTG) and weekly ultrasound scans. Elective cesarean hysterectomy was planned at 38 weeks of gestation. A midline vertical incision was used to provide adequate exposure. A classical cesarean section was performed under general anesthesia with delivery of a healthy female infant weighing 3.0 kg. Total abdominal hysterectomy was performed. The procedure required transfusion of four units of packed red blood cells and four units of FFP. Postoperatively, the patient required five days of intensive care monitoring for hemodynamic stability and fluid balance management, followed by 10 days in the general ward for complete recovery. Histopathology confirmed placenta percreta. She was discharged in good condition with complete recovery and no long-term complications (Figure 4).

Case 6: Emergency Management With Bladder Repair

**Figure 5:**
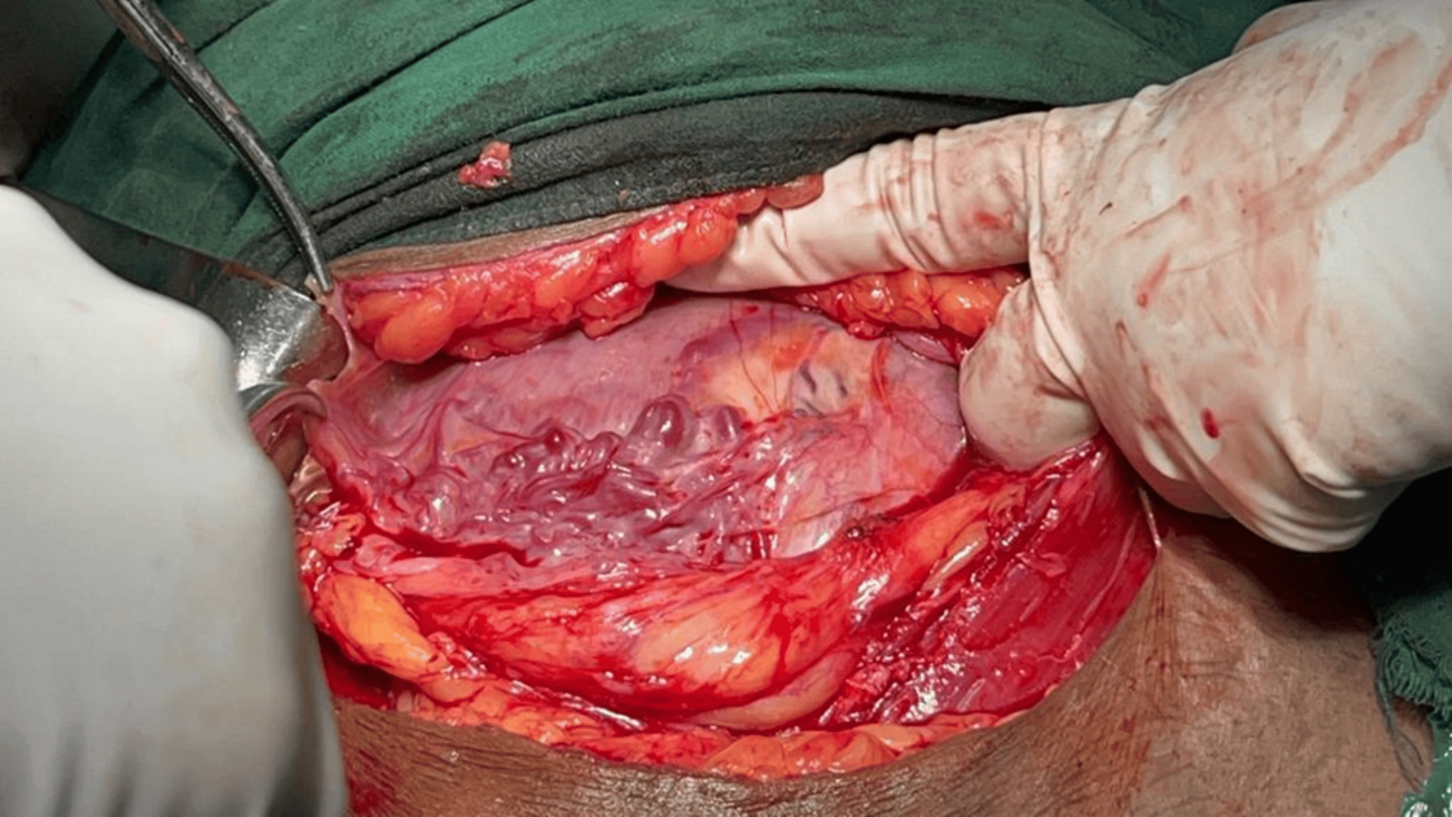
Intraoperative view of extensive uterine vascular network Surgical photograph showing the markedly dilated and tortuous uterine vessels characteristic of placenta accreta spectrum, emphasizing the need for careful vascular control during surgical management

A 34-year-old third gravida with two previous cesarean sections and pregnancy-induced hypertension was referred in active labor at 37 weeks of gestation. Emergency ultrasound revealed reduced myometrial thickness at the cesarean scar site with evidence of placental invasion and increased vascularity (AIP Grade 3). Emergency cesarean section with obstetric hysterectomy was performed under general anesthesia. Intraoperatively, extensive placental invasion into the bladder wall was identified, requiring coordinated urological intervention for bladder wall repair. The extensive vascular network surrounding the uterus was clearly visible. The bladder was repaired in two layers using absorbable sutures. The patient required prolonged catheterization for three weeks to ensure adequate bladder healing. Blood transfusion included four units of packed red blood cells, four units of FFP, and four units of random donor platelets. The patient developed transfusion-related acute lung injury (TRALI), which was managed with supportive care. Histopathology confirmed placenta percreta. Despite the complexity of the case, she recovered well and was discharged in stable condition on postoperative day 12 (Figure 5).

Case 7: Complex Percreta with Multidisciplinary Management

**Figure 6:**
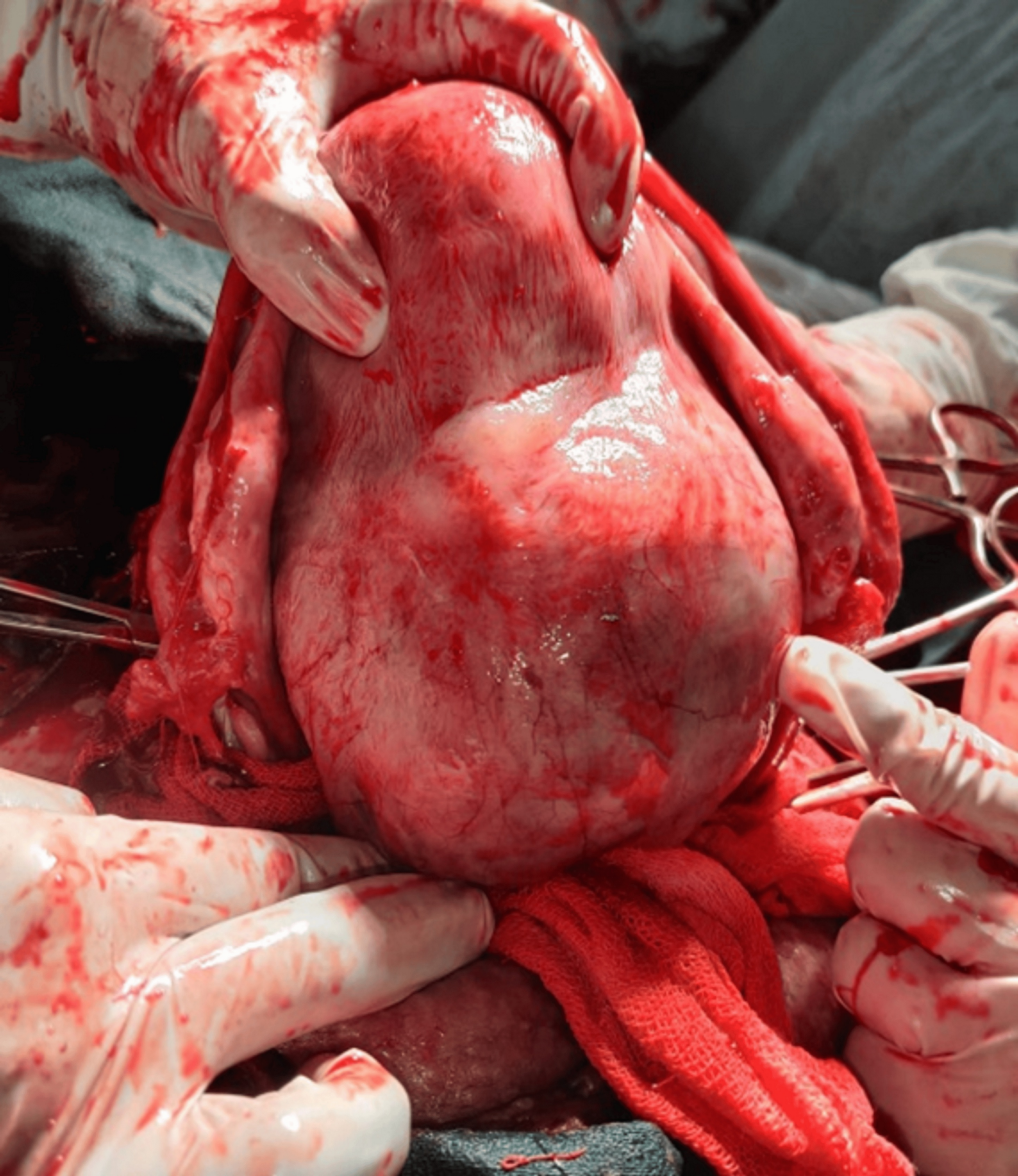
Complex surgical field showing extensive vascular involvement Intraoperative view demonstrating the complex vascular anatomy encountered in placenta percreta cases, with extensive collateral circulation and altered tissue planes requiring experienced surgical expertise

A 36-year-old fifth gravida with three previous cesarean sections and one spontaneous abortion was referred at 36 weeks of gestation with placenta percreta (AIP Grade 3). Comprehensive imaging evaluation included both ultrasound and MRI. MRI demonstrated grade 4 placenta previa with extensive placenta percreta in the anterior lower uterine segment and suspicious bladder adherence without full-thickness invasion. Elective cesarean section with planned hysterectomy and potential bladder repair based on intraoperative findings was performed at 36 weeks. A midline vertical incision was made. The complexity of the case was evident from the extensive placental invasion and altered anatomy. The final surgical specimen demonstrated characteristic features of placenta percreta with deep myometrial invasion extending to the serosal surface. Histopathology confirmed placenta percreta. The patient required two units of packed red blood cells and four units of FFP intraoperatively, with an additional two units of packed red blood cells postoperatively. She required seven days of intensive care monitoring for hemodynamic stability and organ function assessment. Both mother and baby recovered completely and were discharged in good health on postoperative day 14 (Figure 6).

Case 8: Placenta Increta With Emergency Management

A 30-year-old fourth gravida with two previous cesarean sections and one abortion was referred at 36 weeks of gestation for suspected placenta accreta. Ultrasound revealed breech presentation with grade 4 placenta previa and features suggestive of placental adherence. MRI confirmed placenta increta with posterior superior bladder thinning but no frank invasion (AIP Grade 2). The patient developed spontaneous labor pains and was taken for emergency classical cesarean section with planned obstetric hysterectomy. The procedure was performed under general anesthesia with multidisciplinary team support. Histopathology confirmed placenta increta. The patient recovered well with no significant complications and was discharged in good condition.

## Discussion

**Figure 7:**
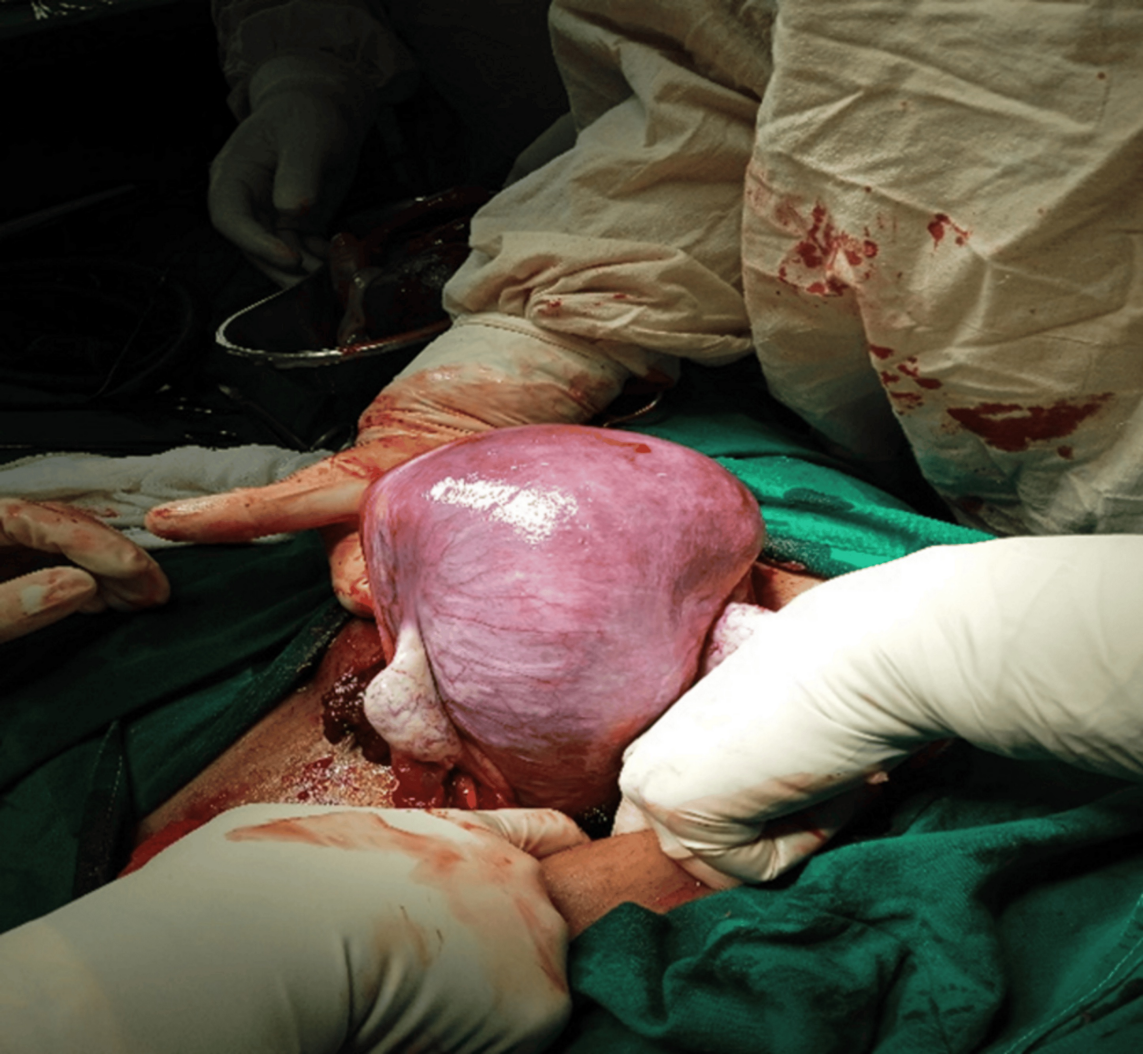
Intraoperative view of placental invasion into bladder wall Surgical photograph showing direct placental invasion into the bladder wall during cesarean hysterectomy, requiring coordinated urological intervention and bladder repair to ensure complete resection

**Figure 8:**
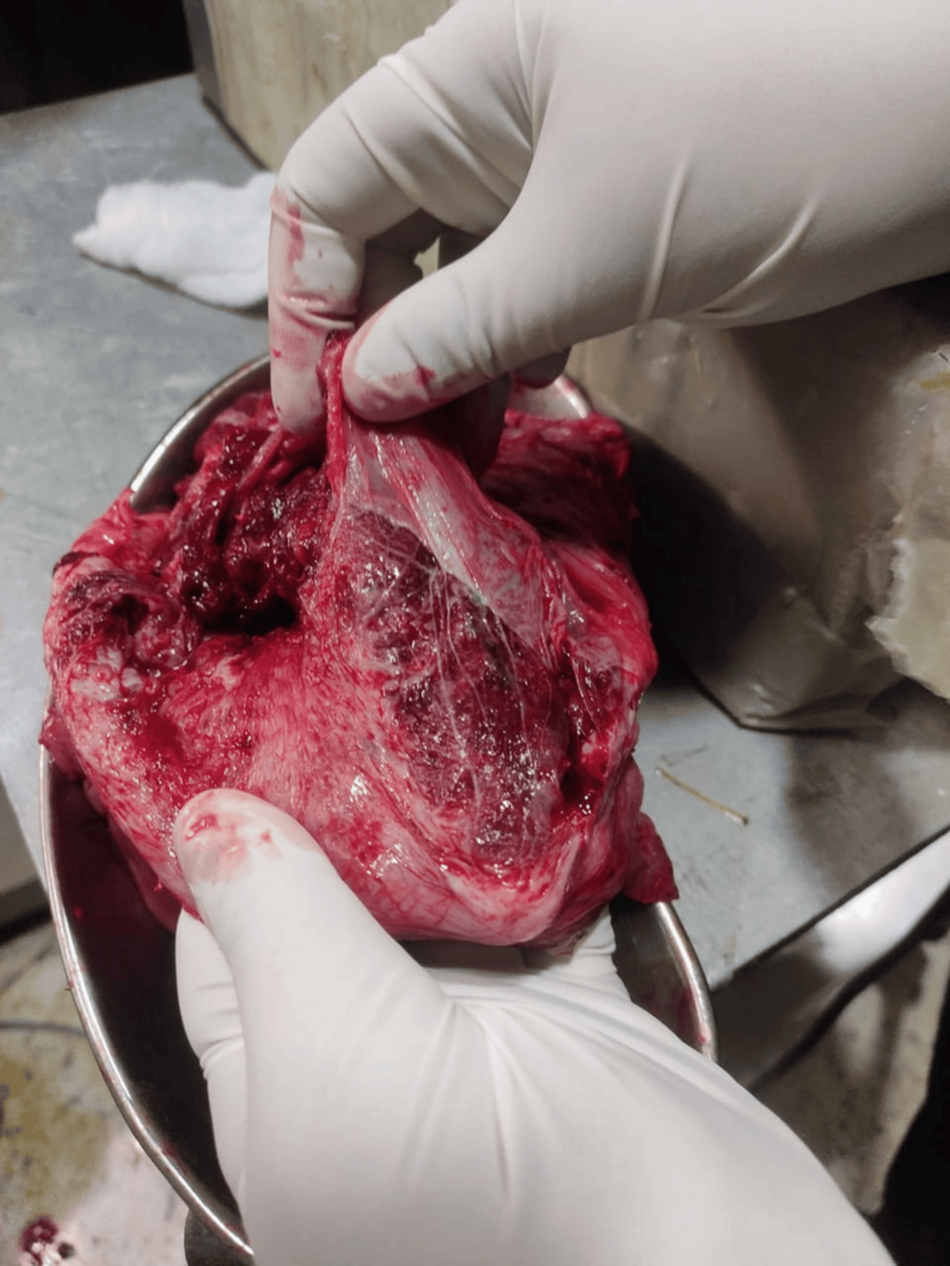
Hysterectomy specimen showing extensive placental invasion Gross pathological specimen of the uterus following cesarean hysterectomy, demonstrating deep placental invasion through the myometrium with areas of placenta percreta, confirming the preoperative diagnosis

This comprehensive case series of eight consecutive PAS cases (Table 1) demonstrates the effectiveness of a standardized multidisciplinary approach in achieving excellent maternal outcomes. The diversity of presentations, from a unique second-trimester termination in a primigravida to complex cases of placenta percreta with bladder invasion, highlights the spectrum of challenges encountered in PAS management and the critical importance of individualized care strategies (Figures 7, 8)

The achievement of zero maternal mortality despite managing several high-risk cases with placenta percreta and bladder invasion reflects the critical importance of early recognition, appropriate referral to a tertiary center, and coordinated multidisciplinary care. Our standardized protocol ensured that all cases were managed by experienced teams with immediate access to blood banking, interventional radiology, and intensive care facilities. The importance of a multidisciplinary team in a center of excellence for PAS is well-established and is a key recommendation in international guidelines [7].

**Figure 9:**
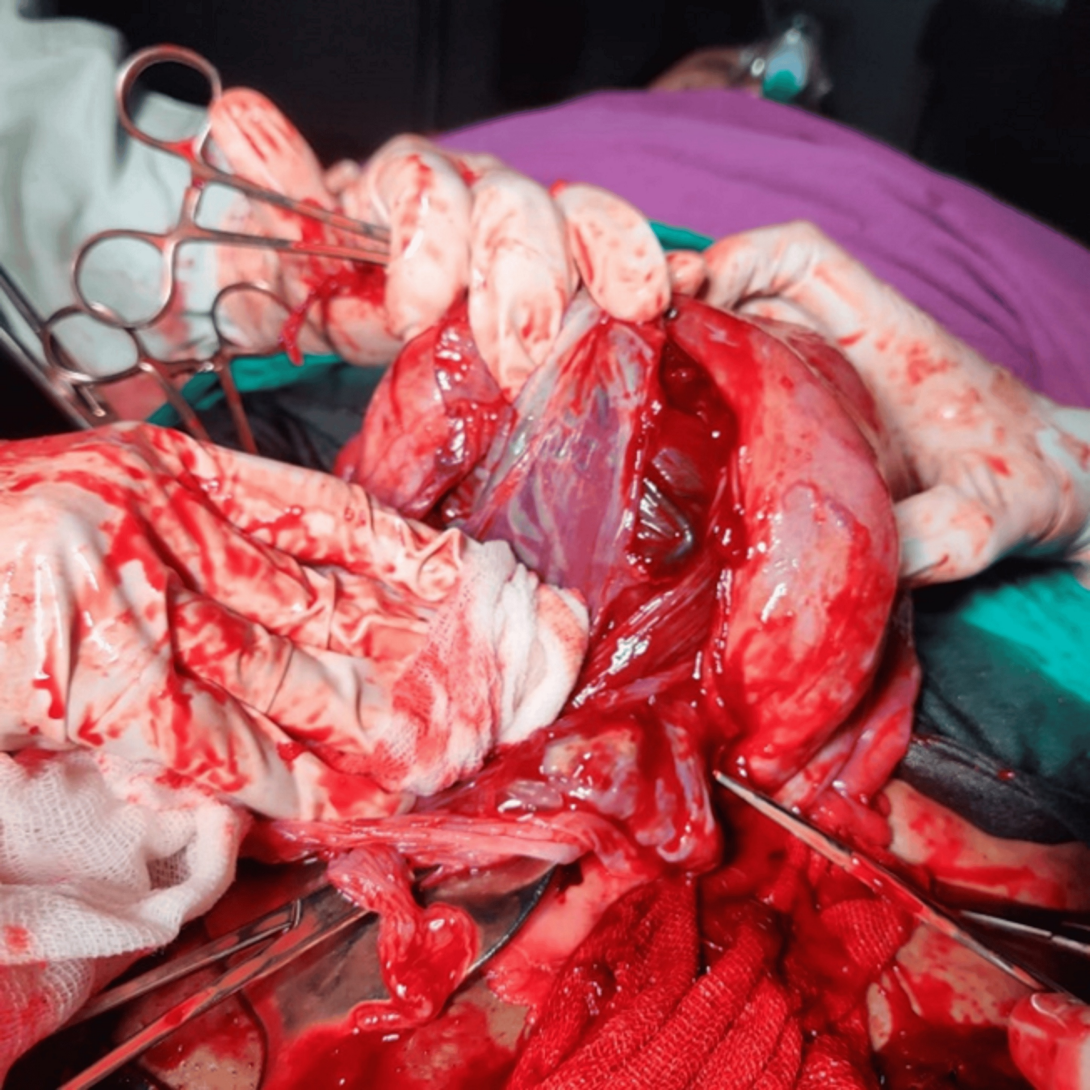
Intraoperative view of placental adherence preventing normal separation Surgical photograph demonstrating firm placental adherence to the myometrium with inability to achieve normal placental separation, necessitating immediate decision for cesarean hysterectomy to prevent catastrophic hemorrhage

The vascular anatomy encountered during these procedures (Figures 6, 9) highlights the complexity of PAS cases and the need for experienced surgical teams familiar with the altered anatomy. The extensive vascular networks, including the characteristic "spaghetti-like" appearance of dilated vessels over the lower uterine segment and bladder serosa, require careful surgical technique and prompt decision-making to minimize blood loss and complications. The recognition of these vascular patterns is crucial for intraoperative decision-making and can guide the surgical approach.

**Figure 10:**
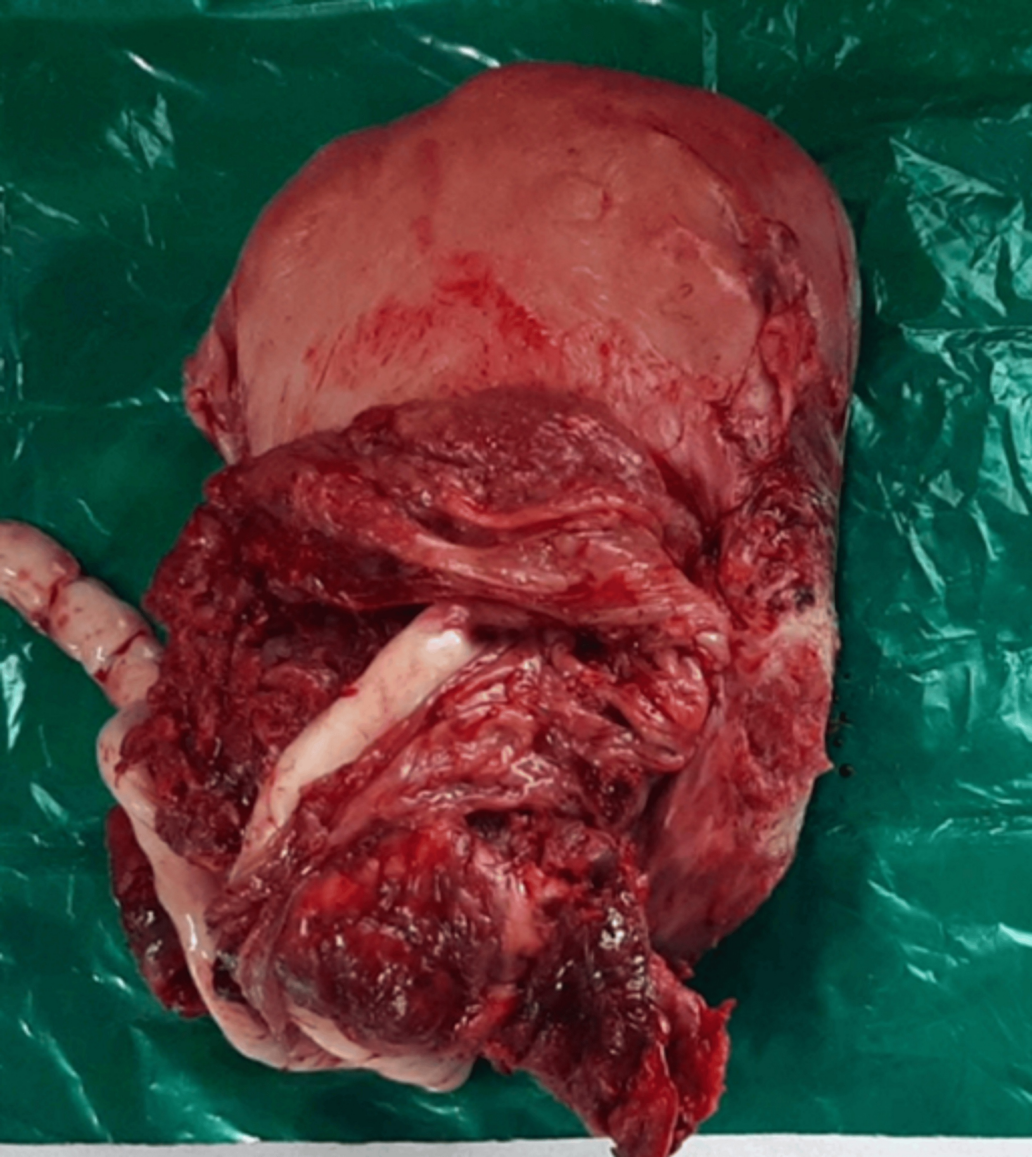
Final surgical specimen demonstrating placenta percreta characteristics Complete hysterectomy specimen showing the characteristic features of placenta percreta with deep myometrial invasion and areas of placental tissue extending through the uterine wall, confirming the severity of the condition

The imaging findings in our series, particularly the ultrasound and MRI characteristics, were crucial for preoperative planning and risk stratification. The sonographic features, including loss of the clear zone, presence of placental lacunae, and increased vascularity, provided essential diagnostic information. MRI findings, particularly the loss of the normal T2 hypointense myometrial border and demonstration of placental invasion depth, were invaluable for surgical planning. The intraoperative findings, including the extensive neovascularization patterns (Figure 5) and varying degrees of placental invasion (Figures 1, 2, 3, 5, 6, 10), provided valuable insights into the pathophysiology of PAS and guided surgical decision-making.

Case 3 represents a particularly innovative approach to PAS management and contributes significantly to the literature on fertility-sparing techniques. The use of prophylactic bilateral UAE in a primigravida with second-trimester placenta increta allowed for successful fertility-sparing surgery with minimal blood loss. The clear demonstration of the placental separation plane following embolization supports the effectiveness of this technique in carefully selected cases [9]. This approach represents a paradigm shift in the management of early-gestation PAS cases where fertility preservation is desired.

The management of placenta percreta with bladder invasion (Cases 4, 5, 6, and 7) presented significant challenges requiring urological expertise and careful surgical planning. The intraoperative findings (Figure 5) clearly demonstrated the extent of bladder involvement, emphasizing the importance of multidisciplinary planning and the need for urological consultation in all cases of suspected placenta percreta. Our approach, including preoperative planning with urology consultation and intraoperative urological support, was crucial in managing these complex cases. The surgical specimens (Figures 5, 10) provided clear evidence of the depth of placental invasion, supporting the histopathological diagnosis and validating the preoperative imaging findings.

Our experience reinforces several key principles in PAS management that are essential for optimal outcomes. First, early antenatal diagnosis through systematic screening of high-risk patients is essential for appropriate planning and referral to specialized centers. Second, delivery should occur in a tertiary care center with comprehensive resources, including blood banking, interventional radiology, and experienced multidisciplinary teams. Third, the decision for cesarean hysterectomy should be made promptly when placental adherence is encountered, as attempts at forceful placental removal invariably lead to catastrophic hemorrhage and increased morbidity. Fourth, conservative management may be appropriate in carefully selected cases, as demonstrated in Case 5, but requires intensive monitoring and immediate access to emergency surgical intervention.

The timing of delivery in our series varied based on clinical circumstances and individual risk assessment, with most elective cases delivered between 36-38 weeks of gestation. This timing represents a careful balance between the risks of preterm delivery against the increasing risk of emergency presentation with advancing gestational age. Our experience supports the current recommendations for planned delivery at 34-36 weeks in cases of placenta previa with suspected PAS, while acknowledging that individual circumstances may require modification of this approach [10].

Blood transfusion requirements varied significantly across cases, reflecting the spectrum of disease severity and the effectiveness of prompt surgical intervention. The cases managed with prophylactic embolization (Case 3) and those with early hysterectomy decisions (Cases 1 and 2) had lower transfusion requirements, supporting the importance of these management strategies in reducing maternal morbidity. The availability of massive transfusion protocols and experienced blood banking support was crucial in managing the more complex cases with significant blood loss.

Imaging modalities for PAS

Our series relied on a combination of ultrasound and MRI for diagnosis and surgical planning. While ultrasound remains the cornerstone of PAS diagnosis, MRI can be a valuable adjunct, particularly in cases of posterior placenta or suspected deep myometrial or organ invasion [1]. However, the utility of routine MRI for all PAS cases is debated. Some studies suggest that high-quality ultrasound by an experienced operator is superior to MRI in diagnosing PAS [3], while others report that MRI improves the detection of cases requiring hysterectomy [4]. Our institutional practice is to use MRI selectively, which aligns with the current evidence suggesting that its routine use may not be necessary in all cases, especially when high-quality ultrasound is available [10].

Uterine artery embolization and fertility preservation

Case 3 represents a particularly innovative approach to PAS management and contributes significantly to the literature on fertility-sparing techniques. The use of prophylactic bilateral UAE in a primigravida with second-trimester placenta increta allowed for successful fertility-sparing surgery with minimal blood loss. This approach is gaining traction as a method to reduce intraoperative hemorrhage and facilitate uterine conservation [7]. The timing of the hysterotomy 24 hours post-embolization was a deliberate decision to allow for ischemic changes to develop, thereby reducing the vascularity of the placental bed and minimizing blood loss. This case highlights the potential of UAE as a key component of conservative management in carefully selected patients who desire future fertility.

Surgical management and the triple-P procedure

While we did not utilize the “triple-P” procedure (perioperative placental localization, pelvic devascularization, and placental non-separation) as a complete package in our series, our management principles align with its core tenets. The triple-P procedure, which involves a combination of surgical techniques to control hemorrhage, is effective in reducing morbidity in PAS cases [8]. Our approach, which included planned cesarean hysterectomy with the placenta left in situ and the use of UAE in one case, reflects a similar emphasis on hemorrhage control. The decision to perform a hysterectomy was made promptly in cases of significant hemorrhage, which is a critical step in preventing maternal exsanguination.

Management of placenta percreta with bladder invasion

The management of placenta percreta with bladder invasion (Cases 4, 5, 6, and 7) presented significant challenges requiring urological expertise and careful surgical planning. Our approach, including preoperative planning with urology consultation and intraoperative urological support, was crucial in managing these complex cases. The use of preoperative ureteric stenting and intraoperative methylene blue dye to confirm bladder integrity is an important strategy to minimize urological complications [10].

Limitations

This study has several limitations. First, the retrospective nature of the case series and the small sample size of eight patients limit the generalizability of our findings and preclude statistical analysis of risk factors and outcomes. Second, as a single-center study conducted at a tertiary care government hospital, our results may not be representative of outcomes in other healthcare settings with different resource availability or patient populations. Third, the lack of standardized follow-up protocols and variable follow-up durations across cases limit our ability to assess long-term outcomes and late complications. Fourth, ultrasound and MRI images of the patients are not available for publication, which limits the ability to demonstrate the imaging characteristics that guided our diagnostic and management decisions. Fifth, the absence of a control group prevents direct comparison of our multidisciplinary approach with alternative management strategies. Finally, the two-year study period may be insufficient to capture the full range of PAS presentations and complications that could emerge over a longer duration of observation. Despite these limitations, this case series provides valuable insights into the multidisciplinary management of PAS and demonstrates that excellent maternal outcomes can be achieved through coordinated care in a tertiary center setting.

## Conclusions

This comprehensive case series demonstrates that excellent maternal outcomes can be achieved in PAS management through a coordinated multidisciplinary approach, early diagnosis, and individualized treatment planning. The diversity of cases presented, including innovative approaches such as prophylactic UAE and conservative management strategies, provides valuable insights for clinicians managing this challenging condition. The achievement of zero maternal mortality across eight consecutive cases, including several with placenta percreta and bladder invasion, underscores the effectiveness of standardized protocols and specialized care in tertiary centers. Future research should focus on refining diagnostic criteria to improve early detection, optimizing timing of interventions based on individual risk factors, and developing evidence-based protocols for conservative management in selected cases. The development of standardized training programs for multidisciplinary teams and the establishment of regional centers of excellence will be crucial for improving outcomes as the incidence of PAS continues to rise globally.
